# Novel FABP4^+^C1q^+^ macrophages enhance antitumor immunity and associated with response to neoadjuvant pembrolizumab and chemotherapy in NSCLC via AMPK/JAK/STAT axis

**DOI:** 10.1038/s41419-024-07074-x

**Published:** 2024-10-01

**Authors:** Dong Zhang, Min Wang, Gen Liu, Xin Li, Wenwen Yu, Zhenzhen Hui, Xiubao Ren, Qian Sun

**Affiliations:** 1 Tianjin Medical University Cancer Institute and Hospital, National Clinical Research Center for Cancer, Tianjin, China; 2 Key Laboratory of Cancer Prevention and Therapy, Tianjin, China; 3 Tianjin’s Clinical Research Center for Cancer, Tianjin, China; 4 Key Laboratory of Cancer Immunology and Biotherapy, Tianjin, China; 5 Department of Immunology, Tianjin Medical University Cancer Institute and Hospital, Tianjin, China; 6 Department of Pathology, Tianjin Medical University Cancer Institute and Hospital, Tianjin, China; 7 Department of Biotherapy, Tianjin Medical University Cancer Institute and Hospital, Tianjin, China

**Keywords:** Non-small-cell lung cancer, Immunotherapy, Tumour immunology

## Abstract

**Abstract:**

Immune checkpoint inhibitors (ICIs) immunotherapy facilitates new approaches to achieve precision cancer treatment. A growing number of patients with non-small cell lung cancer (NSCLC) have benefited from treatment with neoadjuvant ICIs combined with chemotherapy. However, the mechanisms and associations between the therapeutic efficacy of neoadjuvant pembrolizumab and chemotherapy (NAPC) and macrophage subsets are still unclear. We performed single-cell RNA sequencing (scRNA-seq) and identified a novel FABP4^+^C1q^+^ macrophage subtype, which exhibited stronger proinflammatory cytokine production and phagocytic ability. This subtype was found to be more abundant in tumor tissues and lymph nodes of major pathological response (MPR) patients compared to non-MPR patients, and was associated with a good efficacy of NAPC. Multiplex fluorescent immunohistochemical (mIHC) staining was subsequently used to verify our findings. Further mechanistic studies indicated that FABP4 and C1q regulate the expression of proinflammatory cytokines synergistically. In addition, FABP4 and C1q promote fatty acid synthesis, enhance anti-apoptosis ability and phagocytic ability of macrophage via the interaction of AMPK/JAK/STAT axis. This study provides novel insights into the underlying mechanisms and predictive biomarkers of NAPC. Our findings contribute to improving the prognosis of patients with NSCLC by potentially guiding more precise patient selection and treatment strategies.

**Novelty & Impact Statements:**

- We identified a group of macrophages (FABP4^+^C1q^+^ macrophages) related to the therapeutic efficacy of neoadjuvant chemoimmunotherapy. FABP4^+^C1q^+^ macrophages highly expressed proinflammatory cytokines-related genes and had a strong cytokine production and phagocytic ability. We believe that our study provides a novel insight into the synergistic mechanism of neoadjuvant ICI combined with chemotherapy and may lead to improved clinical outcomes in patients with NSCLC in the future.

## Introduction

Lung cancer remains one of the leading causes of cancer-related deaths worldwide. The mortality rate of lung cancer is the highest among all cancers, with an average 5-year survival rate of less than 20%. Despite advances in treatment, the prognosis for non-small cell lung cancer (NSCLC) patients has not significantly improved over the past decades [1]. Current therapeutic strategies, including chemotherapy and radiotherapy, often fail to achieve long-term remission, partly due to the complexity of tumor microenvironment (TME) and immune evasion mechanisms. Understanding the interactions within the TME could lead to the development of more effective immunotherapies and improve survival rates for NSCLC patients.

In recent years, the use of immunotherapy-based regimens combined chemotherapy for advanced NSCLC has significantly ameliorated the prognosis of patients. Moreover, the use of neoadjuvant immune checkpoint inhibitors (ICIs) combined with chemotherapy is usually recommended to decrease tumor bulk and eradicate micrometastases prior to surgery, leading to a better prognosis after curative resection in advanced NSCLC patients [2]. Several clinical trials of neoadjuvant immunotherapy combined with chemotherapy have been proved that chemoimmunotherapy is safe and does not lead to surgical delay in breast cancer [3, 4], squamous cell carcinoma of head and neck [5], and NSCLC [6]. Chemotherapy can achieve antitumor effects by inhibiting tumor proliferation and activating immune responses such as increasing the infiltration of T cells and inducing the expression of favorable immunizing antigens [7, 8]. The key anti-tumor effect of ICIs relies on reversing T cell exhaustion through PD-1/PD-L1 immune checkpoint blockade [9]. Compared to neoadjuvant chemotherapy or immunotherapy, neoadjuvant chemoimmunotherapy achieved more pathological and radiological relief, and has a high surgical resection rate [10, 11]. The combination of ICIs with chemotherapy provided a durable overall survival (OS) benefit in metastatic NSCLC patients compared to chemotherapy alone [12]. However, the specific molecular mechanisms of combination therapy remain unclear. Exploring the synergistic molecular mechanisms of neoadjuvant chemoimmunotherapy and its interaction with the TME is crucial.

The TME is the environment around the tumor, including tumor cells, surrounding blood vessels, myeloid immune cells such as macrophages and dendritic cells, lymphocytes, cytokines, chemokines, and the extracellular matrix [13]. Recent studies have identified enriched effector T cells and NK cells in the tumor after neoadjuvant therapy [14]. Enrichment of CD56^dim^ NK cells, CD8^+^ T cells was also associated with favorable clinical response to neoadjuvant ICIs immunotherapy combined chemotherapy [15, 16]. Nevertheless, there are few studies focused on macrophage subsets in the TME after treatment of neoadjuvant ICIs immunotherapy combined chemotherapy in NSCLC. Recent study revealed M1 macrophage predicted good efficacy of neoadjuvant ICIs combined with chemotherapy. Complete pathological response (CPR) tumors had a stronger pre-established immune infiltrate at baseline than non-CPR, characterized by higher levels of M1 macrophages in NSCLC [17, 18]. Tian et al. proposed that high percentage of CD68^+^PD-L1^+^ macrophages could serve as a promising biomarker for neoadjuvant immunotherapy in combination with other therapies (including targeted therapy and chemotherapy) in head and neck squamous cell carcinoma (HNSCC) [19]. Therefore, it is imperative to explore novel macrophage signature as a biomarker to predict the efficacy of neoadjuvant ICIs immunotherapy combined chemoimmunotherapy.

The present study aims to investigate the changes of macrophage subsets after neoadjuvant pembrolizumab and chemotherapy (NAPC), identify the clinical prognostic value of the novel signature FABP4^+^C1q^+^ macrophages in NSCLC patients and explore their potential role in neoadjuvant chemoimmunotherapy. Our findings could pave the way for novel therapeutic targets and enhance the efficacy of existing chemoimmunotherapy for NSCLC.

## Materials and methods

### Single-cell suspension preparation

We isolated fresh tumors (T), regional draining lymph nodes (LN), distal normal lung tissues (D), and peripheral blood before (P0) and after (P1) neoadjuvant therapy from NSCLC patients for single-cell preparation. Distal normal lung tissues were defined as being located 10–15 cm from the tumor margin of the surgical specimens. Peripheral blood mononuclear cells (PBMCs) were collected using density gradient centrifugation. All biopsy specimens were minced and digested in RPMI 1640 containing tumor tissue dissociation enzyme for approximately 1 h at 37 °C. Then the cells were treated with red blood cell lysis buffer for 2 min to remove red blood cells before filtration through a 40-µm cell strainer. To obtain live cells, dead cell removal microbeads were used according to the manufacturer’s instructions. Next, CD45^+^ immune cells were obtained by positive magnetic cell sorting using CD45 MicroBeads. Cells were counted and stained with 0.4% Trypan blue and adjusted to the cell density of 1000 cells/μL for 10× Genomics single cell suspension sequencing.

### Single-cell capture and scRNA-seq library construction

CD45^+^ immune cells (1000 living cells/μL) were loaded onto a 10× Genomics Chromium^TM^ Single Cell Controller Instrument (10× Genomics, Pleasanton, USA). Single Cell 5’ Library & Gel Bead Kit v1.0 (Cat# 1000006, 10× Genomics) and Single Cell A Chip Kit (Cat# 1000152, 10× Genomics) were used to acquire single-cell gel beads in emulsion according to the manufacturer’s instructions. CD45^+^ immune cells were lysed, and mRNA was released to produce cDNA by reverse transcription in a single gel bead in emulsion. Subsequently, the cDNA was used as a template for polymerase chain reaction (PCR) amplification to construct a cDNA library. Sequencing analysis was performed using the Illumina HiSeq sequencing platform.

### scRNA analysis

Cell Ranger v.3.1.0 (10× Genomics) was used to process raw sequencing data and generate a gene expression matrix. The Seurat R package (version 3.2.1) was used for quality control, downstream analyses, and to determine the major cell subtypes. Uniform manifold approximation and projection (UMAP) was used for dimensionality reduction and cluster visualization. We identified and annotated clusters according to marker genes and displayed the results in a two-dimensional UMAP plane. “Find Markers” and “Find All Markers” functions of Seurat package were used to identify differentially expressed genes (DEGs). Kyoto Encyclopedia Genes and Genomes (KEGG) and Gene ontology (GO) enrichment analysis were applied to analyze the biological process in which DEGs were enriched in each subset.

### Cell culture and transfection

CD14^+^ monocytes were separated by CD14 MicroBeads from human PBMCs. To induce macrophages, CD14^+^ monocytes were treated with complete medium containing 50 ng/ml human macrophage-stimulating factor and cultured at 37 °C with 5% CO2 for 9 days. To obtain M1-macrophges and M2-macrophaegs, 20 ng/ml IFN-γ and 100 ng/ml Lipopolysaccharide or 20 ng/ml IL-4 and 20 ng/ml IL-13 were used for 48 h respectively. A total of 8 × 10^5^ PBMCs-derived macrophages were seeded in 6-well plates and transfected with either 20 nM of specific siRNA or siNC (control) for 48 h. siRNAs were purchased from GenePharma (Suzhou, China). Macrophages were treated with AMPK activator at a concentration of 1 mM and 2 mM for 24 h. The catalog numbers and concentrations of reagents are listed in Supplementary Table 1 (Table S1). The sequences of the siRNAs were provided in Supplementary Table 2 (Table S2).

### Quantitative reverse transcription PCR (RT-qPCR) and western blotting

Total RNA was extracted and reverse-transcribed to cDNA using PrimeScript™ RT Master Mix. β-actin (*ACTB*) was used as an internal reference, and the comparative Ct method (2^−ΔΔCt^) was used to analyze the relative expression levels. The sequences of primers are listed in Table S2.

Proteins from logarithmic phase cells were lysed with RIPA and PMSF (100:1) on ice for 30 min. Protein samples were separated by 10% SDS-PAGE and transferred onto PVDF membranes. The membrane was blocked with a blocking solution for 15 min and incubated with a specific primary antibody at 4 °C overnight. The next day, after incubating with the corresponding secondary antibody, the Image Studio software was used for visualization and analysis. The catalog numbers and concentrations of primary antibodies and reagents are provided in Table S1.

### Flow cytometry (FC)

Single-cell suspensions were prepared for flow cytometry analysis. Cells were blocked using Fc Receptor Blocking Solution for 8 min at room temperature, and then incubated with surface antibody for 30 min in the dark at 4 °C. After cell surface staining, the cells were fixed and permeabilized using the Fixation/Permeablization Kit according to the manufacturer’s instructions. The cells were intracellularly stained and washed twice with washing buffer before FC. FC was performed using an BD-Aria II device (BD Biosciences, San Diego, CA, USA), and data was analyzed with FlowJo software (version 10.1, USA). The antibodies used for FC staining are listed in Table S1.

### Cell cycle, apoptosis, and phagocytosis assessment

Cell cycle and apoptosis assays were performed using fluorescence-activated cell sorting. 1 × 10^6^ cells were fixed in 75% ethanol at −20 °C for 12 h. The next day, the cells were washed with pre-cooled PBS, and stained with PI for 20 min at room temperature before analysis.

The apoptosis assay was performed using Annexin V-APC and PI Apoptosis Kit. Cells were incubated with annexin V-APC and PI for 15 min in the dark at room temperature before flow cytometry analysis.

Phagocytic activity was assessed with pHrodo Deep Red *E. coli* BioParticles. Macrophages (10^5 cells/well) were plated in a 96-well plate with 100 μL of culture medium. After cells were adhered to the plate for 1 h, then incubated with 100 μL pHrodo BioParticles for 30 min at 37 °C. After harvesting and washing, macrophages were fixed with 4% paraformaldehyde and analyzed by flow cytometry within 48 h.

### Clinical patient cohorts

Eleven stage IIIA NSCLC patients from Tianjin Medical University Cancer Institute and Hospital (TMUCIH) were recruited for single-cell RNA sequencing (scRNA-seq). Their clinicopathological characteristics are summarized in Table 1. The clinical validation cohort included 30 NSCLC patients who had not received NAPC, and 30 patients received NAPC with stage IIIA NSCLC. Basic clinicopathological characteristics are shown in Tables 2 and 3. This study was approved by the Ethical Committee of TMUCIH, and all patients provided informed consent. (NO.: bc2020060).

**Table 1:** Clinical parameters of scRNA sequencing cohort.

Group	Patient	Gender	Age	Smoking	Pathological type	Residual viable tumors (%)	PD-L1expression (%)
naïve	P01	male	58	No	LUAD	N/A	N/A
	P02	male	73	Yes	LUSC	N/A	N/A
	P03	male	75	Yes	LUAD	N/A	N/A
	P04	female	65	No	LUAD	N/A	N/A
MPR	P05	female	51	Yes	LUSC	0	0
	P06	male	62	Yes	LUSC	0	30
	P07	male	67	No	LUSC	0	3
	P08	male	59	Yes	LUSC	10	50
non-MPR	P09	male	46	No	LUAD	60	60
	P10	female	66	No	LUAD	80	90
	P11	male	68	Yes	LUSC	95	15

**Table 2:** Clinical parameters of 30 patients with neoadjuvant pembrolizumab and chemotherapy.

Characteristics	MPR	non-MPR
	(*n* = 20)	(*n* = 10)
Age		
<60	6 (30.0%)	2 (20.0%)
≥60	14 (70.0%)	8 (80.0%)
Gender		
Male	18 (90.0%)	8 (80.0%)
Female	2 (10.0%)	2 (20.0%)
Smoking		
Yes	17 (85.0%)	7 (70.0%)
No	3(15.0%)	3 (30.0%)
clinical TNM stage		
IIIA	19 (95.0%)	9 (90.0%)
IIIB	1 (5.0%)	1 (10.0%)
Pathological type		
LUAD	2 (10.0%)	5 (50.0%)
LUSC	15(75.0%)	5 (50.0%)
other	3 (15.0%)	0 (0%)

**Table 3:** Clinicopathological characteristics of 30 naïve patients and their association with the risk score.

Characteristics	All cases	CD68^+^FABP4^+^C1q^+^ macrophages		*p* value	χ^2^
	*n* (%)	Low (%)	High (%)	Fisher’s	
Age					
<60	20 (66.7)	10 (50.0)	10 (50.0)	0.709	0.605
≥60	10 (33.3)	6 (60.0)	4 (40.0)		
Gender					
Male	22 (73.3)	11 (50.0)	11 (50.0)	1.000	1
Female	8 (26.7)	4 (50.0)	4 (50.0)		
Smoking					
Yes	17 (56.7)	9 (52.9)	8 (47.1)	1.000	0.713
No	13 (43.3)	6 (46.2)	7 (53.8)		
LN metastasis					
Yes	26 (86.7)	14 (53.8)	12 (46.2)	0.598	0.283
No	4 (13.3)	1 (25.0)	3 (75.0)		
TNM stage					
IIIA	27 (90.0)	14 (51.9)	13 (48.1)	1.000	0.543
IIIB	3 (10.0)	1 (33.3)	2 (66.7)		
Pathological type					
LUAD	14 (46.7)	8 (57.1)	6 (42.9)	0.715	0.464
LUSC	16 (53.3)	7 (43.8)	9 (56.2)		

### Multiplex fluorescent immunohistochemistry (mIHC)

Opal 7-Color technology Kit was used for mIHC staining according to the instructions. After gradient deparaffinization in xylene, rehydration in ethanol, and antigen retrieval in EDTA (pH = 9.0), paraffin-embedded tissue slides were blocked with Ab-blocking buffer at room temperature for 10 min. Then the slides were incubated with the primary antibody at 4 °C overnight. The next day, slides were incubated with the secondary antibody for 10 min at room temperature. TSA Visualization and signal amplification were achieved with Opal TSA Plus (1:100), and the Ab-TSA complex was removed by heating with EDTA buffer. Multiplex staining was repeated using the above steps, and nuclei were stained with DAPI. Immunostaining was evaluated with the Mantra quantitative pathology imaging system (PerkinElmer). A total of five random fields at ×200 magnification was used to calculate the mean numbers of positive cells by two independent pathologists. The catalog numbers and concentrations of antibodies and reagents are provided in Table S1.

### Statistical analysis

The patient characteristics were described by descriptive statistics. Continuous variables were analyzed using the *t*-test and one-way ANOVA with GraphPad Prism 8.0. Correlations were assessed with Spearman’s analysis. Kaplan–Meier survival analysis was used for prognostic analysis. We selected Cox regression proportional hazard models to quantify hazard ratios (HRs) of death from NSCLC in the univariable analyses. All data are presented as the mean ± SD, Significance was set as *P < 0.05; **P < 0.01; ***P < 0.001; ****P < 0.0001.

## Results

### Single-cell landscape of macrophages and its association with NAPC in NSCLC patients

**Fig. 1:**
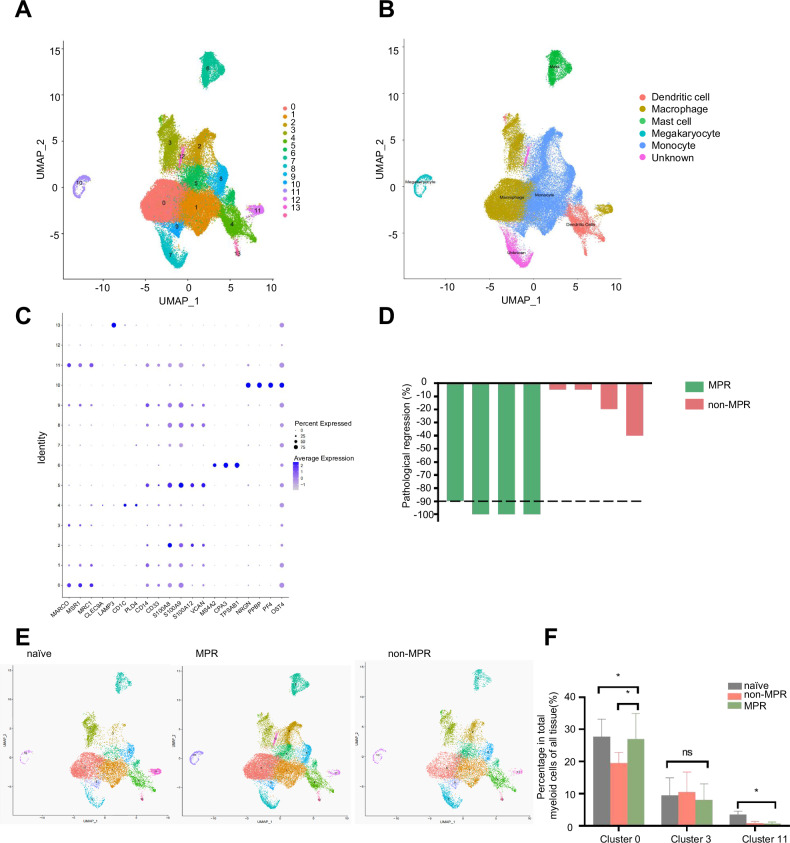
Single-cell landscape of macrophages and its association with neoadjuvant pembrolizumab and chemotherapy (NAPC) in NSCLC patients. **A** Uniform manifold approximation and projection (UMAP) of myeloid immune cells in 11 scRNA-sequenced samples. A total of 14 myeloid cell clusters were identified. **B** Cell-type annotation of myeloid immune cells. **C** Cell-type annotation markers, and the annotation process. MARCO, MSR1, and MRC1 were used as macrophage markers; CLEC9A, LAMP3, CD1C, and PLD4 were used as dendritic cell markers; CD14, CD33, S100A8, S100A9, S100A12, and VCAN were used as monocyte markers; MS4A2, CPA3 and TPSAB1 were used as mast cell markers; and NRGN, PPBP, PF4, and OST4 were used as markers of megakaryocytes. **D** Based on the acquired major pathological response, seven patients with NAPC were divided into two groups (MPR and non-MPR groups). **E** UMAP of myeloid immune cells in the treatment-naïve, MPR, and non-MPR groups. **F** Changes in the proportion of C0, C3, C11 macrophages in total number containing all tissues in treatment-naïve, MPR, and non-MPR groups.

To identify macrophage subsets associated with the clinical efficacy of NAPC, we collected fresh tumor tissues (T), regional draining lymph node (LN), distal normal lung tissues (D), and peripheral blood before (P0) or after (P1) NAPC treatment from 11 patients with stage IIIA NSCLC, and then isolated CD45^+^ immune cells for 10× single-cell RNA sequencing. We included four naïve patients without treatment and seven patients who received NAPC before surgery. After quality control and filtration, we acquired 157,788 immune cells, including 36,891 cells identified as myeloid-derived cells. Additionally, we collected a total of 10,528 cells from tumor tissues, 9283 cells from distal normal lung tissues, 2985 cells from regional draining lymph nodes, 3807 cells from P0, and 10,288 cells from P1. Principal component analysis was applied to cluster data according to the DEGs. Finally, the clustering results were displayed using UMAP. We identified 14 myeloid cell clusters (Fig. 1A). Three macrophage clusters (C0-macrophage-FABP4/C1q, C3-macrophage-ACP5, C11-macrophage-TUBB) were identified. The remaining myeloid-derived cell clusters including five clusters for monocytes (C1-monocyte-CCL3, C2-monocyte-S100A8, C5-monocyte-VCAN, C8-monocyte-THBS1, C9-monocyte-CXCL10), two clusters enriched for dendritic cells (C4-DC- HLA-DPB12, C13-DC-LAMP3), one cluster of mast cells (C6-mast cells-TPSAB1), one cluster for megakaryocyte (C10- megakaryocyte-PPBP), and two unknown cell subsets (C7 and C12) (Fig. 1B, C).

Two senior clinicopathologists evaluated the pathological response and used major pathological response (MPR) as an early alternative endpoint in our study. MPR refers to achieving viable tumor cells ≤10% in resected surgical specimens after surgical resection. The pathological remission of patients in NAPC group was assessed, and patients were divided into two groups: MPR and non-MPR patients (Fig. 1D). To evaluate the relationship between macrophage subtypes and the clinical efficacy of NAPC, we analyzed different cell subsets in the treatment-naïve, MPR and non-MPR groups (Fig. 1E). And the UMAPs of myeloid immune cells in 11 samples were supplied in Fig. S1. We identified that macrophages constituted a large proportion of the whole myeloid-derived immune cells and C0-macrophage cluster changed most significantly in different NAPC efficacy groups. Compared with non-MPR group, the percentage of C0-macrophages in total number contains all tissues of patients was higher in MPR group (Fig. 1F). The proportion of C3-macrophages did not differ significantly in the three different groups. The percentage of C11-macrophages was observed significantly decreased after neoadjuvant therapy. However, C11-macrophages proportion is very low. Therefore, we hypothesized that C0-macrophages play an important role in predicting the clinical efficacy of NAPC.

### C0-macrophages, a novel gene signature macrophage subset: expression of FABP4 and C1q simultaneously with high expression of proinflammatory cytokines

**Fig. 2:**
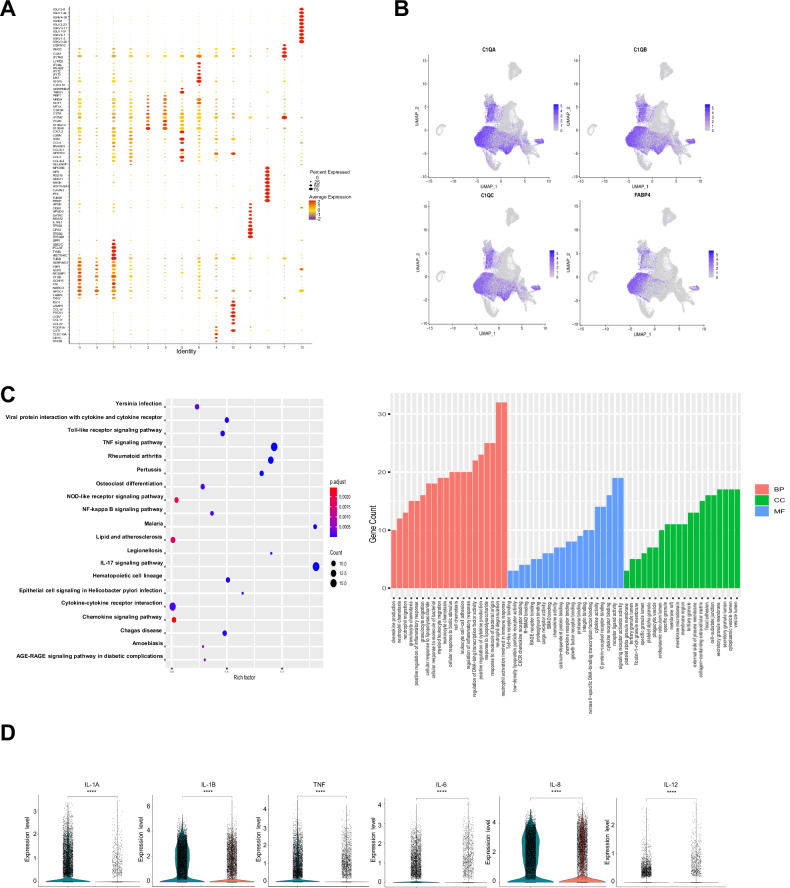
C0-macrophages, a novel gene signature macrophage subset: expression of FABP4 and C1q simultaneously with high expression of proinflammatory cytokines. **A** Identification of novel marker genes in different clusters of myeloid immune cells after sorting the genes by log|Fold Change| and selecting the top ten marker genes. **B** UMAP of the expression of novel marker genes (*FABP4, C1QA, C1QB, C1QC*) of C0-macrophages. **C** Gene Ontology and Kyoto Encyclopedia of Genes and Genomes enrichment analysis of characteristic genes in FABP4^+^C1q^+^ macrophages. **D** The cytokines (IL-1α, IL-1β, TNF-α, IL-6, IL-8, IL-12) expression levels of FABP4^+^C1q^+^ macrophages and other macrophage subsets in our scRNA-seq profile (Blue: C0-macrophages; Red: other macrophages).

To investigate the marker genes of C0-macrophages, the expression of characteristic genes in C0-macrophages were explored. Fatty acid-binding protein 4 (FABP4) and Complement C1q A-Chain (C1QA) were observed as the top two marker genes in C0-macrophage (Fig. 2A, B). FABP4 is an adipokine that plays an important regulatory role in lipid and glucose metabolism. Current research indicates that it enhances the polarization to the proinflammatory M1 subtype [20]. Complement C1q (consisting of C1QA, C1QB and C1QC three subunits) is a complex glycoprotein that mediates a variety of immunoregulatory functions and plays a critical role in the prevention or promotion of tumorigenesis and development [21, 22].

To further explore the biological functions of FABP4^+^C1q^+^ (double-positive, DP) macrophages, DEGs were subjected to GO and KEGG enrichment analysis. FABP4^+^C1q^+^ macrophages were mainly concentrated in the positive regulation of cytokine production and inflammatory response, chemokine production, neutrophil activation, leukocyte chemotaxis, and migration (Fig. 2C). Compared with other macrophages, FABP4^+^C1q^+^ macrophages have higher expression level of cytokines (including IL-1A, IL-1β, TNF-α, IL-6, IL-8, IL-12,) than other macrophages in our scRNA-seq database (Fig. 2D).

### Enrichment of FABP4^+^C1q^+^ macrophages in tumor tissues and lymph nodes indicated good therapeutic efficacy of NAPC and good prognosis in NSCLC

**Fig. 3:**
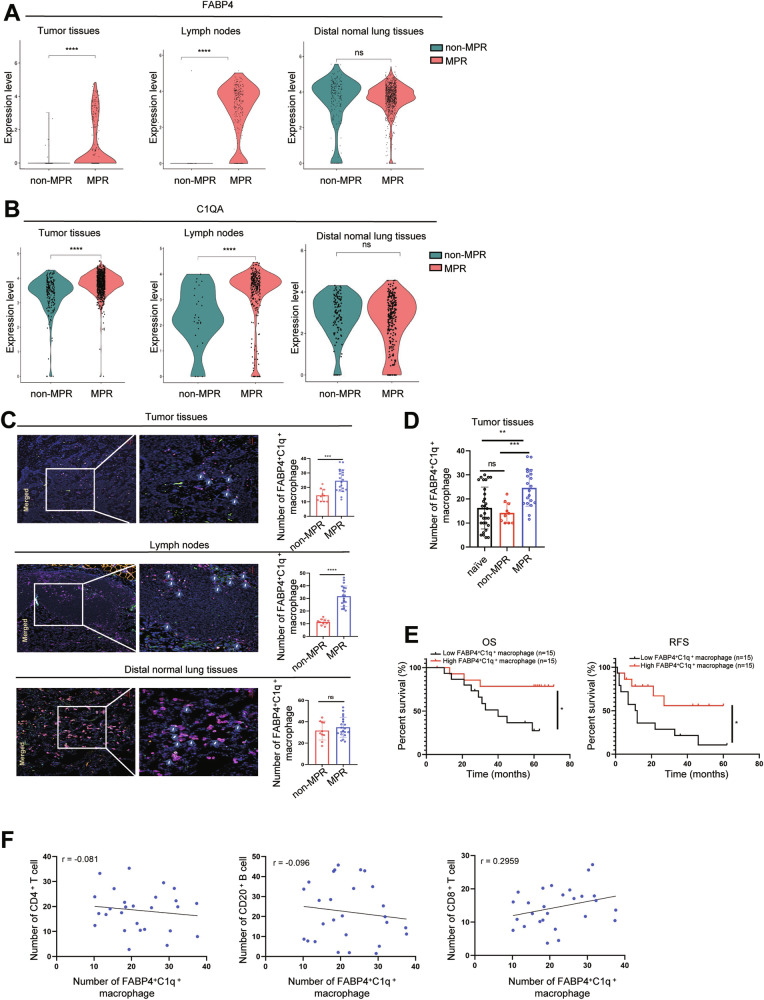
Enrichment of FABP4^+^C1q^+^ macrophages in tumor tissues and lymph nodes indicated good therapeutic efficacy of NAPC and good prognosis in NSCLC. **A**, **B** Comparison of FABP4 and C1QA expression levels in tumor tissues (T), lymph nodes (LN), distal normal tissues (D) of MPR and non-MPR groups. **C** Representative mIHC staining of NSCLC tumor tissues, lymph nodes, distal normal lung tissues showing CD68 (purple), C1q (green), and FABP4 (orange). Original magnification: ×200. Scale bar: 50 µm. **D** The number of FABP4^+^C1q^+^ macrophages infiltration in tumor tissues among naïve, MPR, non-MPR NSCLC patients. **E** Analysis of correlation between the level of tumor infiltrate FABP4^+^C1q^+^ macrophages (low and high) with OS and RFS in NSCLC, using Kaplan–Meier survival analysis. **F** Correlation between the number of tumor infiltrate FABP4^+^C1q^+^ macrophages and CD4^+^ T cells, CD20^+^ B cells, CD8^+^ T cells.

Next, to evaluated the clinical significance of FABP4^+^C1q^+^ macrophages on NAPC therapeutic efficacy, the expression levels of FABP4 and C1QA were analyzed in different tissue types of neoadjuvant MPR, and non-MPR patients using scRNA-seq profile. We found that the expression of FABP4 and C1QA were significantly higher in tumor tissues (T) and lymph nodes (LN) in MPR than in non-MPR patients. There was no difference in the expression of FABP4 and C1QA in distal normal lung tissues (D). This result indicates that FABP4^+^C1q^+^ macrophages play a critical role in the TME (Fig. 3A, B). Subsequently, 30 patients received NAPC were assessed (Table 2). Multiplex fluorescent immunohistochemistry (mIHC) of FABP4, C1q, and CD68 (a common surface marker of macrophages) was performed on paraffin-embedded tumor tissues, lymph nodes, distal normal lung tissues of patients with NSCLC, and the same results were obtained (Fig. 3C). In addition, the infiltration of FABP4^+^C1q^+^ macrophages were also higher than naïve patients with no treatment before surgery (Fig. 3D).

Next, to explore the clinical prognostic value of FABP4^+^C1q^+^ macrophages, 30 naïve patients with NSCLC with follow-up data for prognostic evaluation were further studied. The clinicopathological characteristics of patients were presented in Table 3. Statistical analyses indicated that the number of FABP4^+^C1q^+^ macrophages was not significantly correlated with age, gender, smoking, LN metastasis, TNM stage, and pathological type. Overall survival (OS) and recurrence-free survival (RFS) are frequently used to evaluate the prognosis and quality of life for tumor patients. Median expression level of FABP4^+^C1q^+^ macrophages was used as the cutoff value to distinguish between a high-infiltrating group and a low-infiltrating group. Kaplan–Meier survival analysis confirmed that a high level of FABP4^+^C1q^+^ macrophages was associated with longer RFS (*p* = 0.042) and OS (*p* = 0.029) (Fig. 3E). The above results demonstrate that FABP4^+^C1q^+^ macrophages were related to good therapeutic efficacy of NAPC and good prognosis in NSCLC.

Moreover, to investigate the potential interactions between FABP4^+^C1q^+^ macrophages and other immune cells in tumor microenvirment (TME), IHC staining was performed for CD4^+^ T cells, CD8^+^ T cells, CD20^+^ B cells. We observed a clear positive correlation between FABP4^+^C1q^+^ macrophages and CD8^+^ T cells (Fig. 3F).

### FABP4^+^C1q^+^ macrophages have stronger proinflammatory cytokines secretion ability, phagocytic functions, and anti-apoptosis ability

**Fig. 4:**
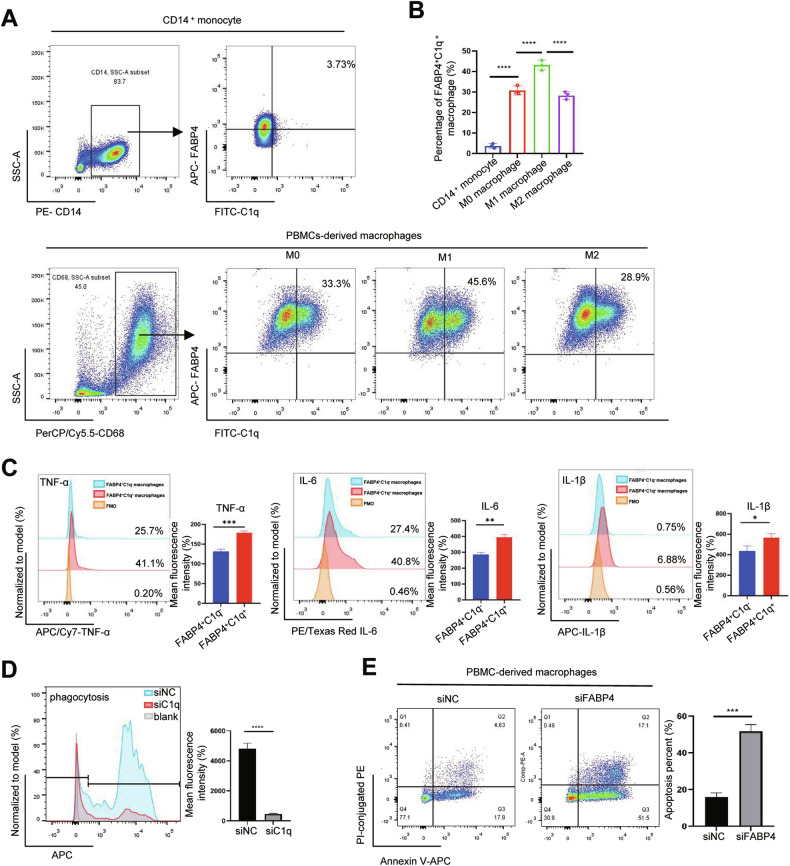
FABP4^+^C1q^+^ macrophages have stronger proinflammatory cytokines secretion ability, phagocytic functions, and anti-apoptosis ability. **A**, **B** The proportion of FABP4^+^C1q^+^ macrophages in CD14^+^ monocytes, M0, M1, and M2 macrophages from human PBMCs. **C** The production levels of inflammatory cytokines (TNF-α, IL-6, IL-1β) in FABP4^+^C1q^+^ and FABP4^+^C1q^−^macrophages (*n* = 3). **D** The phagocytic ability of negative control and C1q knockdown macrophages (*n* = 3). **E** Knockdown of FABP4 in PBMCs-derived macrophages increased cell apoptosis (*n* = 3).

In order to investigate cell functions of FABP4^+^C1q^+^ macrophages, PBMC was used to isolate CD14^+^ monocytes by immunomagnetic beads and induces for macrophages in vitro. Low proportion of FABP4^+^C1q^+^ cells was detected in CD14^+^ monocytes, (Fig. 4A). The proportion of FABP4^+^C1q^+^ cells was increased after inducing to macrophages, especially M1-macrophages (Fig. 4B). We found that proinflammatory cytokines (TNF-α, IL-6, IL-1β) secretion ability of FABP4^+^C1q^+^ macrophages were stronger than that of FABP4^+^C1q^−^ macrophages (Fig. 4C). Moreover, FABP4^+^C1q^+^ macrophages have strong phagocytosis and anti-apoptosis ability. Knocking down the expression of C1q or FABP4 of macrophages, the phagocytic ability or anti-apoptosis functions of the macrophage were significantly weakened respectively (Fig. 4D, E). In conclusion, FABP4^+^C1q^+^ macrophages have strong pro-inflammatory cytokines secretion ability, phagocytic functions, and anti-apoptosis ability. This may be one of the mechanisms of antitumor effect of in FABP4^+^C1q^+^ macrophages.

### FABP4 and C1q regulate the expression of inflammatory cytokines in FABP4^+^C1q^+^macrophages synergistically

**Fig. 5:**
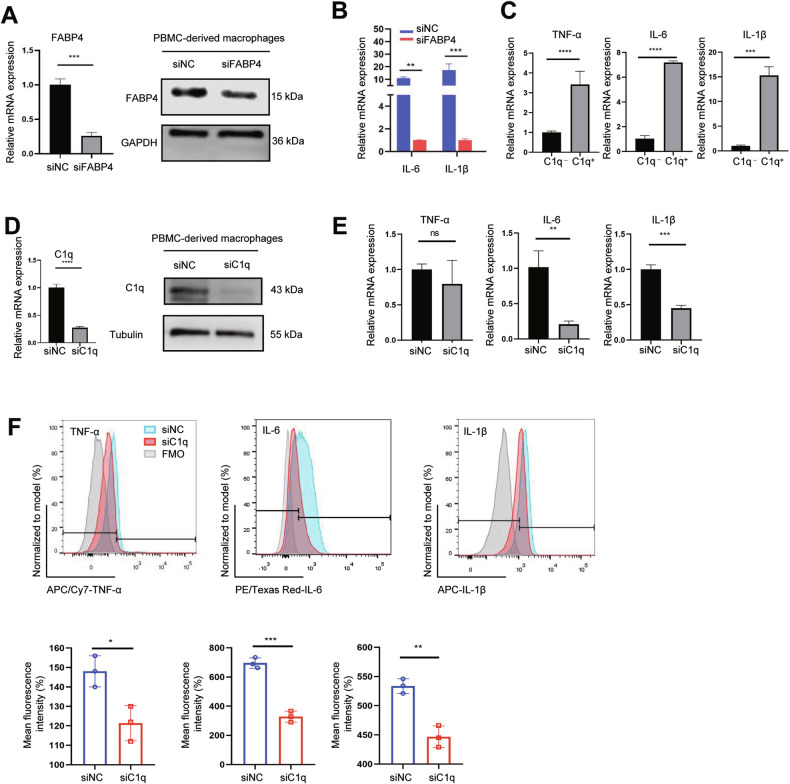
FABP4 and C1q regulate the expression of inflammatory cytokines in FABP4^+^C1q^+^macrophages synergistically. **A** The efficiency of FABP4 knockdown in PBMCs-derived macrophages (*n* = 3). **B** The mRNA expression levels of IL-6 and IL-1β in siNC and siFABP4 macrophages. **C** The mRNA expression levels of proinflammatory cytokines (TNF-α, IL-6, IL-1β) in FABP4^+^C1q^+^ and FABP4^+^C1q^−^ PBMCs-derived macrophages (*n* = 3). **D** The efficiency of C1q knockdown in macrophages detected by RT-qPCR and western blot (*n* = 3). **E**, **F** The proinflammatory cytokines (TNF-α, IL-6, IL-1β) expression and production ability in siNC and siC1q macrophages determined using RT-qPCR and flow cytometry (*n* = 3).

To further explore the regulatory mechanism of FABP4^+^C1q^+^ macrophages inflammatory cytokines expression, we performed the FABP4 knockdown (siFABP4) and C1q knockdown (siC1q) in PBMC-derived macrophages and confirmed the knockdown efficiency using RT-qPCR and western blot (Fig. 5A, D). FABP4 knockdown led to the reduction of IL-6 and IL-1β synthesis of macrophages (Fig. 5B). Furthermore, we found that the expression levels of proinflammatory cytokines (TNF-α, IL-6, IL-1β) in C1q^+^ macrophages were higher (Fig. 5C). Our results indicated that proinflammatory cytokines (including TNF-α, IL-6, IL-1β) decreased after knockdown of C1q on macrophages detected by RT-qPCR and flow cytometry (Fig. 4E, F). The above results indicated that FABP4 and C1q are involved in upstream regulation of proinflammatory cytokines.

### AMPK phosphorylation level decreased in FABP4^+^C1q^+^macrophages

**Fig. 6:**
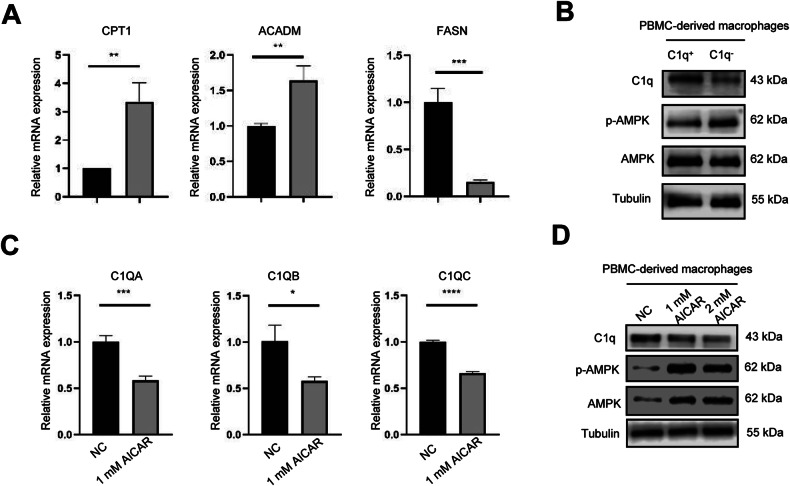
AMPK phosphorylation level decreased in FABP4^+^C1q^+^macrophages. **A** The mRNA expression levels of fatty acid synthase (FASN), and fatty acid lyase (CPT1, ACADM) in siNC and siFABP4 macrophages (*n* = 3). **B** The protein levels of AMPK, and pAMPK in FABP4^+^C1q^+^ and FABP4^+^C1q^−^ PBMCs-derived macrophages (*n* = 3). **C** The mRNA expression levels of C1QA, C1QB, C1QC of macrophages treated with AMPK activator. **D** The protein levels of C1q, AMPK, and pAMPK from PBMC-derived macrophages after treatment with AMPK activator (AICAR) (*n* = 3).

In addition, as a key regulator in lipid metabolism, we further explored the fatty acid metabolism in FABP4^+^C1q^+^ macrophages. FABP4 knocking down in macrophages leading to a decreased of fatty acid synthase (FASN), and an increasing of fatty acid lyase, including CPT1 (Carnitine Palmitoyltransferase 1), ACADM (Acyl-Coenzyme A Dehydrogenase) (Fig. 6A). This result indicates an increase in FABP4^+^macrophage fatty acid synthesis, coupled with the involvement in extracellular fatty acid uptake of FABP4, which all increase FABP4^+^macrophage fatty acid levels, while the elevated fatty acid levels inhibit AMPK phosphorylation. Similarly, we found that The AMPK phosphorylation level of FABP4^+^C1q^+^ macrophages is lower than that of FABP4^+^C1q^-^ macrophages (Fig. 6B). Then macrophages were treated with AMPK activator to elevated AMPK phosphorylation level, we found C1q expression at the transcription level and protein level were decreased (Fig. 6C, D). The above results suggest that the increase of fatty acid levels in FABP4^+^C1q^-^ macrophages promotes AMPK dephosphorylation, and thereby increasing C1q expression.

### FABP4^+^C1q^+^macrophage exert secretion of inflammatory cytokines and phagocytic function through the AMPK/JAK/STAT3 pathway

**Fig. 7:**
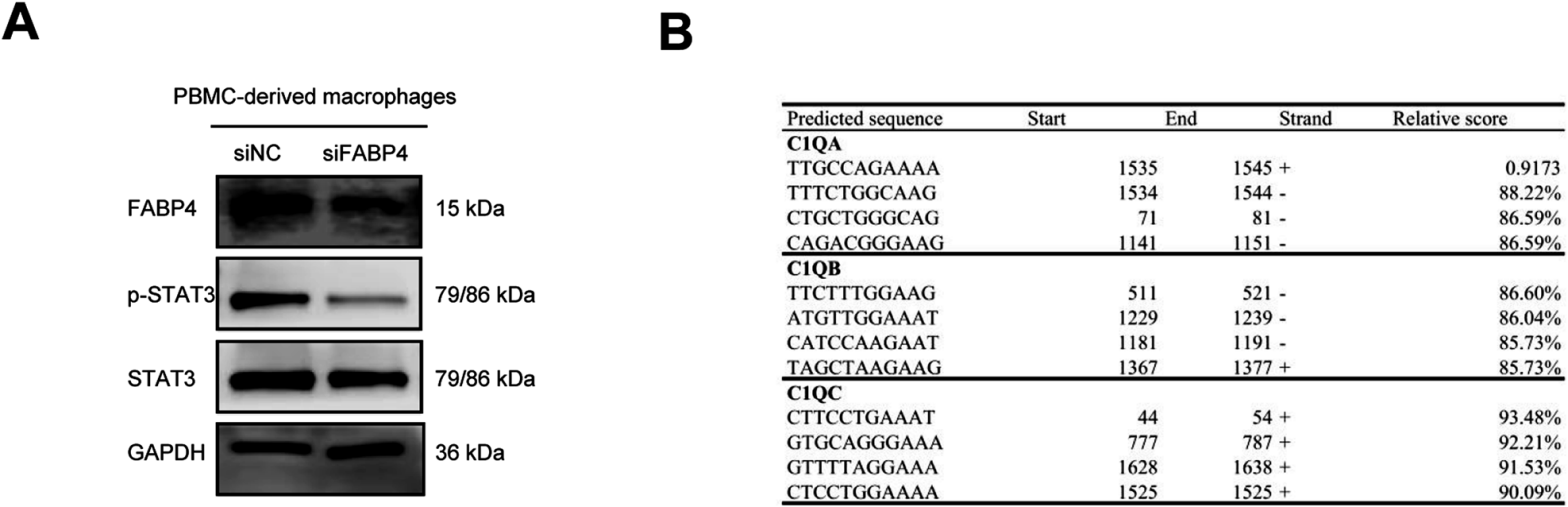
FABP4^+^C1q^+^macrophage exert secretion of inflammatory cytokines and phagocytic function through the AMPK/JAK/STAT3 pathway. **A** The protein levels of STAT3, and pSTAT3 in siNC and siFABP4 groups of macrophages (*n* = 3). **B** C1q subunit gene (C1QA, C1QB, C1QC) promoter region potential STAT3 binding sites, displaying the top four binding sites in the score.

STAT3 is an important transcription factor that regulates the expression of inflammatory cytokines, and we found that JAK2/STAT3 pathway was the most significant signaling in FABP4^+^C1q^+^ macrophages using scRNA-seq profile analysis. We found phosphorylated STAT3 decreased in siFABP4 macrophages (Fig. 7A). Furthermore, we found that there are 19, 24, and 21 promoter regions of C1QA, C1QB, and C1QC genes within 2000 bp respectively with STAT3 binding sites, with a score greater than 80% using JASPAR database analysis (Fig. 7B). In conclusion, AMPK dephosphorylation can relieve the inhibition of JAK2/STAT3 pathway, we speculate that FABP4^+^C1q^+^ macrophages exhibited antitumor effect may through the AMPK/JAK2/STAT3 signaling pathway by regulating inflammatory cytokines expression, phagocytic ability, and anti-apoptosis ability.

## Discussion

Lung cancer, currently ranked first in global cancer-related morbidity and mortality, has 20% of patients are still diagnosed at an advanced stage. Neoadjuvant chemoimmunotherapy is usually a better treatment in advanced NSCLC patients [23–25]. The immune infiltration of TME has become an important factor affecting the efficacy of neoadjuvant chemoimmunotherapy. To date, numerous studies have focused on the variations in the lymphocyte landscape of the TME in NSCLC [26–29]. However, only a few studies have reported the effects of neoadjuvant chemoimmunotherapy, or the underlying mechanisms, on macrophages in NSCLC. In our study, we elaborated on the landscape of macrophages after NAPC, and identified a group of FABP4^+^C1q^+^ macrophages with strong cytokine production and phagocytic ability, which were positively correlated with the success of NAPC in NSCLC and with a better prognosis in patients with NSCLC.

Acting as a molecular microscope for high-throughput sequencing, scRNA-seq is particularly suitable for studying the characteristics of the TME [30]. Our study used scRNA-seq to represent a novel investigation into the role of FABP4^+^C1q^+^ macrophages in the TME, particularly focusing on their prognostic value in NSCLC patients following neoadjuvant chemoimmunotherapy. To our understanding, this study sheds light on the crucial immune role played by FABP4^+^C1q^+^ macrophages and their correlation with favorable therapeutic responses and prognosis among this patient group. Our scRNA-seq results showed that FABP4^+^C1q^+^ macrophages positively correlated with the efficacy of NAPC. FABP4^+^C1q^+^ macrophages were more abundant in MPR patients than in non-MPR patients, especially in tumor tissues and lymph nodes. In general, macrophages infiltrating the tumor are considered to be a double-edged sword, as they have both antitumor and pro-tumor capacities, which are associated with the balance of a series of signals, including cytokines, chemokines, and checkpoints [31]. This FABP4^+^C1q^+^ macrophage subset highly expressed cytokine-related genes and had a strong cytokine production ability. Moreover, high level of FABP4^+^C1q^+^ macrophages was associated with better clinical outcomes in NSCLC patients.

Previous studies revealed FABP4 is mainly expressed in macrophages and adipose tissue, in addition to its involvement in lipid metabolic processes and inflammatory diseases. Additionally, studies have demonstrated that it also participates in the development of various cancers [32–34]. Moreover, recent research on the effect of FABP4 in macrophages has shown that macrophages expressing FABP4 often induce inflammation and play an important role in the development of atherosclerosis, COVID-19, and cancers [35, 36]. These studies demonstrate that FABP4 plays an important role in regulating the production of cytokines in macrophages, which is concordant with our results. Additionally, as a regulator of lipid metabolism, FABP4 regulates fatty acid synthesis and apoptosis in macrophages in our study. A previous study proposed that FABP4 suppresses hepatocellular carcinoma cell proliferation and invasion in vitro and decreases tumor volume in vivo, suggesting that FABP4 could be a potential therapeutic target for cancer [37]. Our results indicated that FABP4, as a biomarker of novel macrophages, can predict the response to NAPC in patients with NSCLC.

The complement system plays an important role in the innate immune system in fighting against pathogens and tumors. C1q is a complex glycoprotein that mediates the clearance of infections and tumor cells, and is important in the classical pathway of the complement system [38]. Moreover, recent studies have shown that C1q is a novel prognostic biomarker that plays different roles in various cancers [39]. It is a pattern recognition molecule that is locally synthesized by macrophages and dendritic cells. Similar to FABP4, the complement system has been suggested to be a double-edged sword that plays a critical role in both the prevention and promotion of tumorigenesis and tumor development [40]. C1q limits tumor progression in a murine cancer model, and has an antitumor effect [41]. However, C1q promotes tumor cell proliferation and migration, and aggravates tumor progression in malignant pleural mesothelioma [42]. It is reported that macrophages are key effectors in the process of complement system activation. According to our in vivo validation experiments, C1q mainly enhances cytokine production and phagocytic ability of macrophages. The presence of macrophages with cytokine production ability is often considered a sign of the activation of the immune system after the use of neoadjuvant ICIs immunotherapy, which is associated with positive clinical outcomes [43]. In addition, previous studies have reported that C1q plays a protective role in the early stage of inflammation development, and then in the late stage of inflammation, and can aggravate disease progression by activating the classical complement pathway [44]. Therefore, moderate C1q activation may be crucial for the antitumor effect of macrophages.

Our study elaborates that FABP4^+^C1q^+^ macrophages are the therapeutic reactive macrophages of NAPC, which have stronger proinflammatory cytokine production ability, phagocytic ability, and anti-apoptotic ability. One of the main limitations of our study is the absence of in vivo validation experiments. To address this, we are actively planning and executing animal studies for in vivo confirmation. Secondly, our study was conducted with a relatively small cohort of patients, which may limit the generalizability of our findings. Future studies with larger and more diverse patient populations are necessary to confirm the robustness and applicability of our results. While we have identified potential therapeutic targets, further translational research is required to develop and test these targets in clinical settings.

In conclusion, our study provides a novel insight into the synergistic mechanism of neoadjuvant ICI combined with chemotherapy and may lead to improved clinical outcomes in patients with NSCLC in the future.

## Supplementary information

Figure S1

Table S1

Table S2

Supplementary Figure and Table Legends

Original Wb

## Data Availability

The study relevant data present in the manuscript. Request for data, experiment protocols or any other questions can be made available to sunqian923@126.com or renxiubao@tjmuch.com.
