# Supplementary figures and images for: Novel FABP4+C1q+ macrophages enhance antitumor immunity and associated with response to neoadjuvant pembrolizumab and chemotherapy in NSCLC via AMPK/JAK/STAT axis
Source: Cell Death Dis. 2024 Oct 1;15(10):717. doi: 10.1038/s41419-024-07074-x (PMC11445384; doi:10.1038/s41419-024-07074-x)

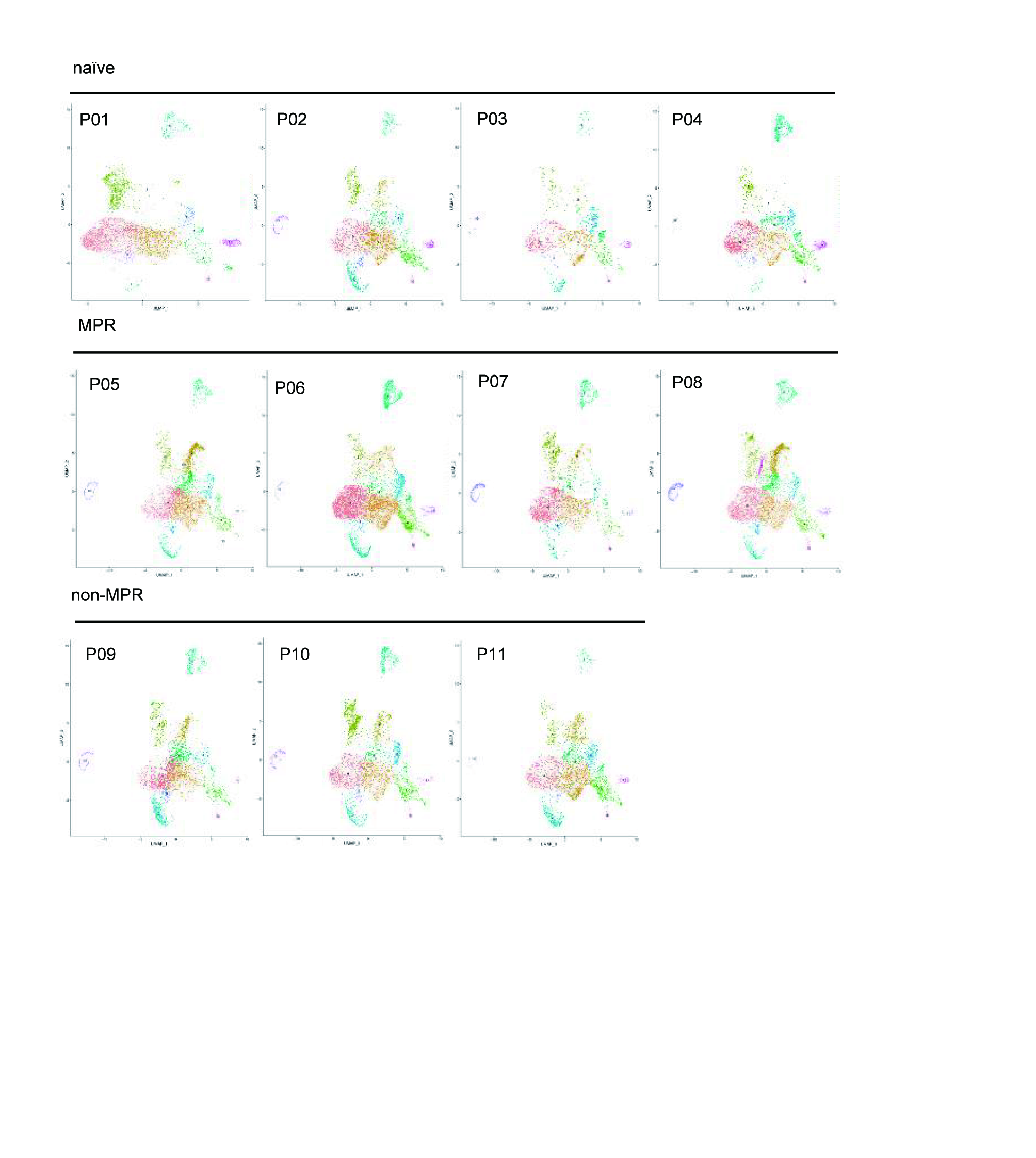

Supplement: Supplementary file 1 — Figure S1 [file 41419_2024_7074_MOESM1_ESM.tif]

**Fig5A**

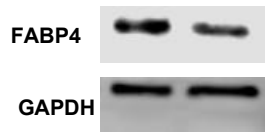

**Fig5D**

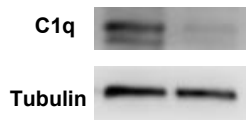

**Fig6B**

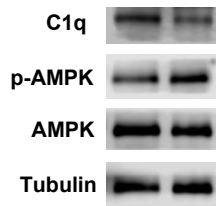

**Fig6D**

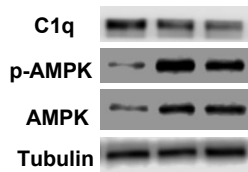

**Fig7A**

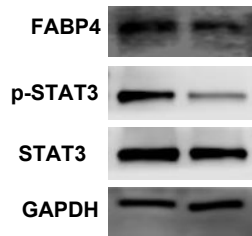

Supplement: Supplementary file 5 — Original Wb [file 41419_2024_7074_MOESM5_ESM.pdf]
