# Supplementary material for: Novel FABP4+C1q+ macrophages enhance antitumor immunity and associated with response to neoadjuvant pembrolizumab and chemotherapy in NSCLC via AMPK/JAK/STAT axis
Source: Cell Death Dis. 2024 Oct 1;15(10):717. doi: 10.1038/s41419-024-07074-x (PMC11445384; doi:10.1038/s41419-024-07074-x)
Supplement: Supplementary file 4 — Supplementary Figure and Table Legends [file 41419_2024_7074_MOESM4_ESM.docx]

**Supplementary Figure and Table Legends**

**Supplementary Figure 1.** UMAP of myeloid immune cells in 11 scRNA-sequenced samples.

**Supplementary Table 1.** Antibodies and reagents used for mIHC, WB, FC, RNA-seq, and cell culture.

**Supplementary Table 2.** The sequence of primers, and siRNAs.
